# DNA Methylation Signatures of the Plant Chromomethyltransferases

**DOI:** 10.1371/journal.pgen.1006526

**Published:** 2016-12-20

**Authors:** Quentin Gouil, David C. Baulcombe

**Affiliations:** Department of Plant Sciences, University of Cambridge, Cambridge, United Kingdom; Gregor Mendel Institute of Molecular Plant Biology, AUSTRIA

## Abstract

DNA methylation in plants is traditionally partitioned into CG, CHG and CHH contexts (with H any nucleotide but G). By investigating DNA methylation patterns in trinucleotide contexts in four angiosperm species, we show that such a representation hides spatial and functional partitioning of different methylation pathways and is incomplete. CG methylation (mCG) is largely context-independent whereas, at CHG motifs, there is under-representation of mCCG in pericentric regions of *A. thaliana* and tomato and throughout the chromosomes of maize and rice. In *A. thaliana* the biased representation of mCCG in heterochromatin is related to specificities of H3K9 methyltransferase SUVH family members. At CHH motifs there is an over-representation of different variant forms of mCHH that, similarly to mCCG hypomethylation, is partitioned into the pericentric regions of the two dicots but dispersed in the monocot chromosomes. The over-represented mCHH motifs in *A. thaliana* associate with specific types of transposon including both class I and II elements. At mCHH the contextual bias is due to the involvement of various chromomethyltransferases whereas the context-independent CHH methylation in *A. thaliana* and tomato is mediated by the RNA-directed DNA methylation process that is most active in the gene-rich euchromatin. This analysis therefore reveals that the sequence context of the methylome of plant genomes is informative about the mechanisms associated with maintenance of methylation and the overlying chromatin structure.

## Author Summary

Dense cytosine DNA methylation (mC) in eukaryotes is associated with closed chromatin and gene silencing. In plants it is well known that the sequence context of the mC (either mCG, mCHG or mCHH) provides a clue as to which of several mechanisms is involved but now, based on detailed analyses of the DNA methylome in wild type and mutants of four plant species, we reveal that there is additional information in the mC sequence context. Low mCCG and over-representation of mCAA and mCTA or mCAT in *A. thaliana* and tomato differentiates regions of the chromosomes near the centromere where methylation is dominated by chromomethyltransferases from the chromosome arms in which mCHH is context-independent and predominantly RNA-directed. Rice and maize have similar sequence context-dependent DNA methylation but the corresponding chromosome domains are not spatially separate as in the dicots. The discovery of the subcomponents of plant methylomes based on sequence context will allow greater resolution in past and future analyses of plant methylomes.

## Introduction

Methylation of cytosine residues plays important roles in gene regulation and transposon control in nuclear genomes of plants and animals. In both plant and animal genomes the methylation is highest on symmetric CG dinucleotides but also exists in CH contexts in which H is any base other than G [1–3]. This non-CG methylation is best characterised in plants where it is normally classified as CHG and CHH contexts [1, 4]. Corresponding to these patterns of DNA methylation in *Arabidopsis thaliana* the maintenance DNA methyltransferases MET1 and Chromomethyltransferase 3 (CMT3) are responsible for the symmetric CG and CHG contexts, respectively [4]. The CMT2 methyltransferase and the small RNA-guided Domains Rearranged Methylase (DRM)1/2 act at the non-symmetric CHH cytosines [5, 6].

There are, however, at least four lines of evidence for additional complexity in the nuclear genome methylome beyond the CG, CHG and CHH components. First, the original whole-genome methylation profiles at base resolution in *A. thaliana* highlighted the possible influence of the local sequence context beyond CG, CHG or CHH on the extent of DNA methylation [7, 8]. Second, in *Physcomitrella patens* and *A. thaliana*, methylation of the CCG trinucleotide context depends on both MET1 and CMT3 orthologs whereas CAG and CTG methylation only requires CMT3 [9]. Third there is an effect of chromatin so that heterochromatic CHH methylation is dependent on CMT2 whereas euchromatic motifs are methylated by small RNA-guided DRM1/2 [5, 10, 11]. The fourth evidence is from humans in which mCH is enriched for various nucleotide motifs depending on the tissue type [3].

To explore these potential methylome complexities we undertook a comprehensive reanalysis of methylation in trinucleotide contexts in *A. thaliana*, maize (*Zea mays*) and rice (*Oryza sativa*). We also analysed the genome-wide methylation in RdDM mutants of tomato (*Solanum lycopersicum*) that we generated by gene editing. We reveal that, at CHG motifs, the methylome is depleted for CCG relative to CAG or CTG throughout the chromosomes of maize and rice and in the pericentric heterochromatin of *A. thaliana* and *S. lycopersicum* where these marks are densest. In the CHH methylome there are also differences between the arms and pericentromere. The euchromatin component is maintained predominantly by the RNA-directed DNA methylation (RdDM) pathway and it is not affected by variations in the sequence motifs adjacent to the C. In the heterochromatin, in contrast, the CHH methylation is densest at CAA and CTA in *A. thaliana* and maize, at CAA and CAT in tomato, and at CTA in rice. This differential CHH subcontext methylation is caused by chromomethyltransferases including CMT2 in *A. thaliana* and ZMET2 and ZMET5 in maize. We also provide evidence that different members of the SUVH H3K9 methyltransferase family impact the differential methylation of CCG compared to CAG and CTG. Based on these findings we propose that analyses of plant DNA methylomes are more informative if they account for subcategories of the mCHG and mCHH motifs. CCG should be considered separately of other CHG contexts and CHH should be subdivided into CAA/CTA and different subcontexts depending on the species.

## Results

### The effect of sequence context on chromosome-wide DNA methylation

**Fig 1:**
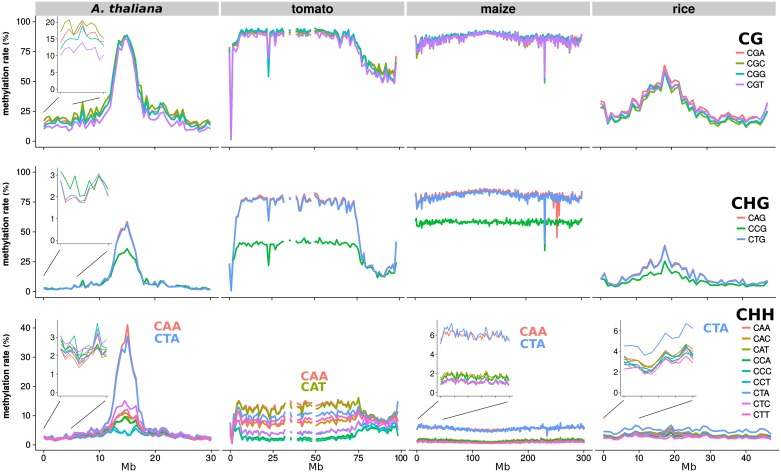
DNA methylation in trinucleotide contexts on chromosome 1 of *A. thaliana* (Col-0) [13], tomato (*S. lycopersicum* cv. M82), maize (*Z. mays* B73) [14], and rice (*O. sativa* indica) [15] leaves. All chromosomes of these plant species are shown in S1–S4 Figs.

The DNA methylation levels in *A. thaliana*, tomato, maize and rice (Fig 1 and S1, S2, S3 and S4 Figs) varied greatly between species and chromosomal regions in CG, CHG and CHH contexts, as previously documented [7, 8, 10, 12]. In *A. thaliana*, there is a relatively small heterochromatic region around the centromere with highly methylated CG. In tomato the chromosomes have short gene-rich arms with 60% mCG and large gene-poor pericentric heterochromatin where CG methylation reaches 85%. Maize chromosomes do not have the same spatial partitioning as their longer and transposon-rich chromosomes appeared uniformly heterochromatic at this scale. Rice is intermediate with a pericentromeric hypermethylated CG region that was more localised than in maize but less so than in *A. thaliana*.

The nucleotide 3’ of CG motifs correlated with small differences in the level of CG methylation in some species (Fig 1). In tomato and *A. thaliana*, CGT methylation was lower than for the other subcontexts and in rice CGA and CGT were generally more methylated than CGG and CGC. In maize, CG methylation was mostly independent of the subcontext. These differences in CG subcontexts were most obvious over genes and transposons (S5 Fig). In *A. thaliana*, CGA methylation was lower than CGC and CGG only in the body of transposons. However in all cases the variations in CG subcontext methylation were much smaller than the context effects described below at CHG and CHH.

As with CG methylation the levels of CHG methylation varied between the heterochromatin and euchromatin (Fig 1) but, unlike CG methylation, there was a large effect of sequence context. In all four plant species CCG methylation was 20-50% lower than CAG/CTG, at least in heterochromatin. In *A. thaliana* and tomato the subcontexts were indistinguishable in the chromosome arms (euchromatin), whereas the CCG methylation remained lower throughout the chromosome in maize and rice.

The CHH context encompasses nine trinucleotide subcontexts. In all species studied here there were major differences between these subcontexts but to a varying extent (Fig 1). *A. thaliana* CHH methylation was low in the arms (2%) without marked differences between subcontexts (Fig 1 and S1 Fig). In the pericentric region, however, methylation of CAA and CTA subcontexts attained 35–40%, whereas CCC and CCT methylation remained below 8% (Fig 1 and S1 Fig). These differences could not be attributed to variations in bisulfite conversion rate because the unmethylated chloroplast showed no such effects (S1 Table). Tomato CHH methylation also differed in the euchromatin and heterochromatin: all sequence contexts had intermediate methylation levels in the euchromatin that were higher (8%) than in *A. thaliana*. In the heterochromatin they were highest (14%) at CAA and CAT and lowest (1–2%) at CCA and CCC (Fig 1 and S2 Fig). In maize the CAA and CTA contexts were the most highly methylated (5–6% versus 2% for other contexts, Fig 1 and S3 Fig and there was no clear CHH differentiation of the pericentric region and the chromosome arms. In rice the methylation at the CTA subcontext (but not CAA) was generally highest (5–6%, Fig 1 and S4 Fig). Non-CG methylation in humans is also influenced by context in a tissue-specific manner, as previously described (S6 Fig, [3]). Contrary to plants, however, there are no chromomethyltransferases in human and CHG methylation is not higher than CHH methylation [4].

To rule out mapping artifacts as the cause of the differential CHG and CHH subcontext methylation we verified that there was no anomaly in sequence coverage along the *A. thaliana* chromosomes and that the profiles were similar if perfect alignment of the sequence data with the genome was required (S7 Fig). We could also rule out that enrichment of CAA and CTA motifs in methylated heterochromatic regions (e.g. transposable elements) could have influenced the profiles (S8 Fig). Finally we eliminated the possibility that specific demethylation of certain CHG and CHH contexts could account for the sequence context effects, based on the similar distribution of context methylation in the *A. thaliana* wild type and the triple DNA demethylase mutant *rdd* (*ros1/dml2/dml3*) (S9 Fig). Our conclusion, therefore, is that the differential CHG and CHH subcontext methylation is affected by properties of the DNA methylation machinery.

### SUVH5/6 rather than SUVH4 regulate CCG methylation in *A. thaliana*

**Fig 2:**
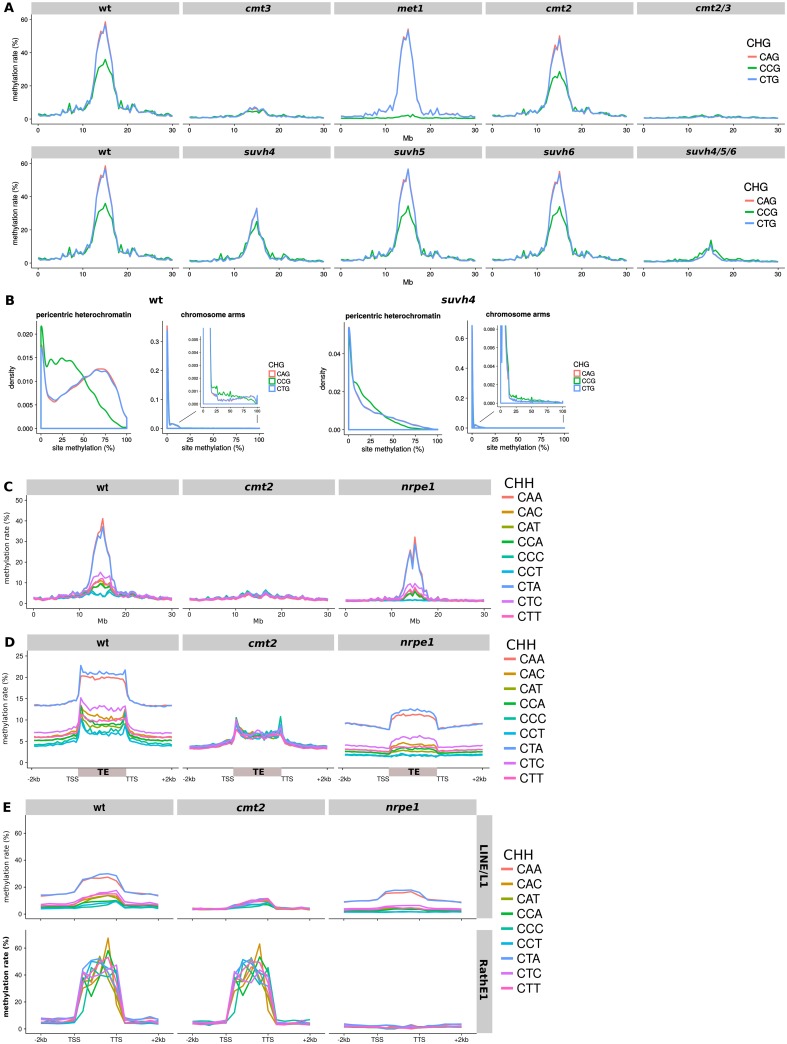
DNA methylation in trinucleotide contexts in *A. thaliana* mutants. (A) CHG methylation along chromosome 1. (B) Distribution of per-site methylation levels in CHG subcontexts in the pericentric heterochromatin (13–16 Mb) and arms (0–10 and 20–30 Mb) of chromosome 1. (C) CHH methylation along chromosome 1. (D) Average CHH methylation along transposable elements. (E) Average methylation over two transposon families: LINE/L1 and RathE1. Other transposon families are shown in S12 Fig. TSS, transcriptional start site; TTS, transcription termination site.

As determined by Yaari et al. [9], the *met1* mutation in *A. thaliana* caused the specific loss of CCG methylation, across the chromosome (Fig 2A). This previous analysis did not show, however, that CCG methylation is lower than CAG/CTG methylation, or that this subcontext effect is more pronounced in the heterochromatin.

The lower CCG methylation was not due to fewer sites being methylated, but instead to the methylated sites having lower levels of methylation (Fig 2B). Efficient maintenance of CHG methylation would normally result in any particular site being consistently either methylated or unmethylated. In agreement with this idea the CAG and CTG sites indeed exhibited a bimodal distribution of methylation at individual sites in both the pericentric heterochromatin and euchromatin of the *Arabidopsis* chromosomes (Fig 2B). CCG methylation, however, had a different pattern with most sites presenting low to intermediate levels of mC (Fig 2B). This suggested that CCG methylation is qualitatively different from CAG/CTG methylation.

The chromomethyltransferases did not influence this subcontext-specific pattern. In the *cmt3* mutant the pericentric region lost 80–90% of its methylation in all three CHG subcontexts whereas the *cmt2* mutation caused a global 25% decrease in CHG methylation in all subcontexts (Fig 2A and S10 Fig). A *cmt2/3* double mutant (Fig 2A) had an even more drastic loss of CHG methylation than *cmt3* but, consistent with the single mutants, it was not a subcontext-specific effect.

We reasoned that the lower CCG methylation could instead be due to a differential recruitment of CMT3 at CCG/CGG versus CAG/CTG duplexes. In that scenario we predicted that SUVH4, SUVH5 and SUVH6 may be involved because they influence H3K9 dimethylation in a positive feedback loop with CHG and CHH methylation [4, 10, 13, 16–18].

Consistent with that idea the *suvh4* mutation disproportionately affected CAG/CTG rather than CCG methylation in the pericentric heterochromatin (Fig 2A and S10 Fig), while all subcontexts remained at near-wild-type levels in the *suvh5* and *suvh6* mutants. Based on this finding we propose that SUVH5 and SUVH6, but not SUVH4, are redundantly able to bind mCGG after replication and thus maintain H3K9 dimethylation of nucleosomes in proximity with CGG/CCG sites leading to recruitment of CMT3 and CCG methylation. SUVH4, in contrast, would recruit CMT3 and thereby mediate mC maintenance at CAG/CTG sites. This hypothesis is supported by the distribution of methylation at individual cytosines in the *suvh4* mutant, where CAG and CTG methylation lost the bimodal profile and resembled CCG methylation (Fig 2B). To explain that the *suvh4* mutation also impacted CCG methylation (but to a lesser extent, Fig 2A and 2B and S10 Fig), we propose that at nucleosomes in proximity with both CAG/CTG and CCG sites, H3K9 dimethylation via SUVH4 binding of CAG/CTG would enhance CMT3 recruitment to the nearby CCG sites.

### CMT2 and RdDM at CHH contexts in *A. thaliana* and tomato

The differential methylation of CHH subcontexts was most pronounced in the pericentric regions in *A. thaliana*, and was due to both higher methylation levels at individual CAA/CTA sites and an increased proportion of sites being targeted for methylation, relative to the other contexts (S11 Fig). We hypothesised that these sites would be affected by the CMT2 pathway, specific to heterochromatin [5, 10], rather than RdDM that is euchromatic [6]. To test this model we analysed published data for *A. thaliana* and maize mutants [13, 14] and new data from tomato RdDM mutants that we generated by gene-editing. Mutation of *CMT2*, leaving only RdDM to methylate CHH, should eliminate the different context effects whereas they would remain in RdDM mutants.

The *A. thaliana* data support the hypothesis because the *cmt2* mutant had reduced but similar methylation of all CHH subcontexts in the pericentromere and over transposons (Fig 2C and 2D), whereas mutation in the major subunit of Pol V (*nrpe1*) in the RdDM pathway left the differential CHH context methylation intact, with elevated CAA and CTA methylation (Fig 2C and 2D and S10 Fig). Closer inspection revealed that the context-independent RdDM affected CHH methylation on the edges of transposons (Fig 2D) where small RNAs accumulate [5]. Furthermore the methylation profiles of two transposon families [19] demonstrated that LINE non-LTR retrotransposons on average exhibit a CMT2-dependent, RdDM-independent methylation profile with elevated CAA and CTA methylation, whereas RathE1 retrotransposons are methylated in an unbiased RdDM-dependent, CMT2-independent fashion (Fig 2E), extending previous results [5]. Whether transposon methylation was CMT2-dependent, RdDM-dependent, or a mixture of both did not depend on the class (I or II) of the transposon family (S12 Fig): it is more likely to be influenced by the distribution of these elements in hetero- and eu-chromatic regions.

**Fig 3:**
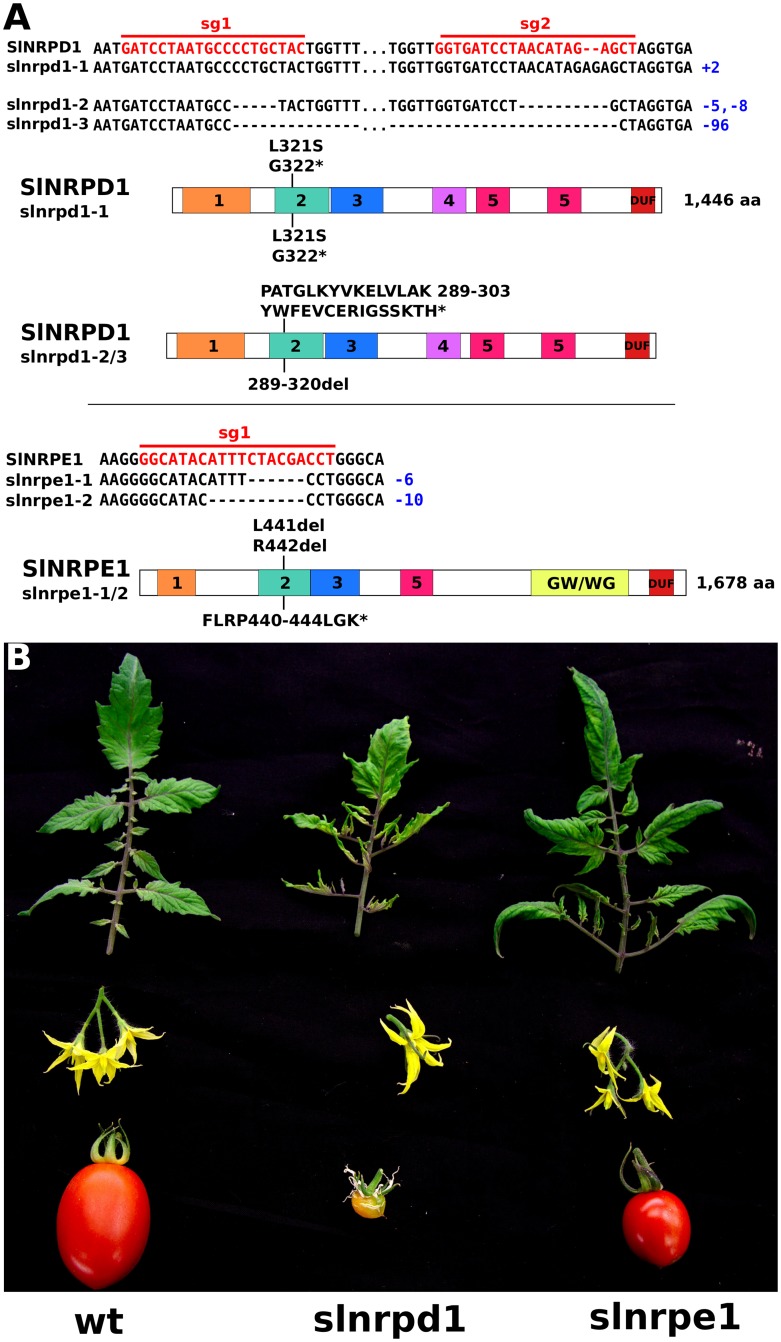
Genotype (A) and phenotype (B) of tomato *slnrpd1* and *slnrpe1* mutants used in this study. The sequences of the sgRNAs (sg1 and sg2) guiding CRISPR-mediated gene editing are indicated in red. Protein domains predicted by HMMER are depicted: domains 1–5 correspond to RNA polymerase Rpb1 domains 1–5; DUF, domain of unknown function.

To evaluate the contribution of RdDM to the CHH methylation profile in tomato we generated mutants of the major subunits of Pol IV and Pol V by CRISPR-Cas gene editing. *SlNRPD1* and *SlNRPE1* are single-copy genes and orthologs of *A. thaliana* AtNRPD1 and AtNRPE1, respectively encoding the major subunits of Pol IV and Pol V. We targeted these genes with pairs of sgRNAs expressed with Cas9 in stable transformants and, among the regenerated plants, several carried a mutation on at least one allele. There were however differences between target genes: while 8 out of 12 plants transformed with constructs targeting *SlNRPD1* carried putative null mutations on both alleles, only 2 out of 11 *SlNRPE1*-targeted plants had both alleles edited. One of these plants died rapidly after transfer to soil, while the other had epinasty, purple pigmentation of old leaves, abnormal flowers, and rare and small fruit despite bearing a likely hypomorphic allele (Fig 3A and 3B). These observations suggested that null mutations of *SlNRPE1* are lethal. All the *slnrpd1* mutants exhibited the same abnormal leaves, flowers and sterility (Fig 3B). The exact correspondence between phenotype and genotype argued against any significant effect from off-target mutations and we selected two *slnrpd1* null mutants and the viable *slnrpe1* hypomorph for further characterization.

**Fig 4:**
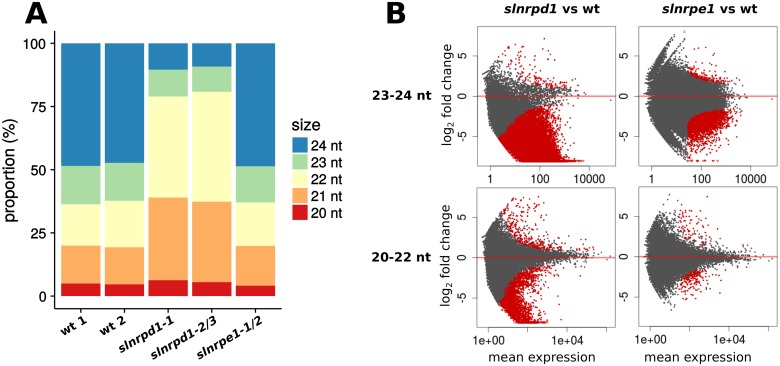
Conserved functions of tomato *SlNRPD1* and *SlNRPE1* in 24-nt sRNA biogenesis. (A) Size profile of small RNA populations in wt, *slnrpd1* and *slnrpe1* (two wt and *slnrpd1* replicates, one *slnrpe1* sample). (B) MA-plot of 23–24-nt and 20–22-nt sRNA loci in wt versus *slnrpd1* and *slnrpe1*. Loci whose sRNA accumulation differed significantly (adjusted p-value < 0.05) between the wild-type and mutant line are plotted in red.

Consistent with the functions of their *A. thaliana* orthologs, mutations of *SlNRPD1* led to a dramatic reduction in 24-nt small RNAs (Fig 4A), whereas the sRNA population profile of *slnrpe1* was similar to wild-type. Correspondingly there was down-regulation of 72% of the 23–24-nt loci with sufficient counts for differential analysis in *slnrpd1* and 13% in *slnrpe1* (Fig 4B). Upregulation was a minor component in both mutant datasets accounting for 0.07% of loci in *slnrpd1* and 0.35% in *slnrpe1*. These tomato data confirm that, as in *A. thaliana* [20, 21], Pol IV is required for the biogenesis of most 23–24-nt siRNAs and Pol V only at a small subset of these loci.

**Fig 5:**
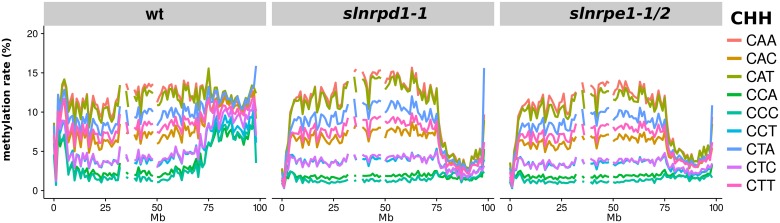
Decreased CHH methylation in the chromosome arms of tomato RdDM mutants. Chromosome 1 is shown.

The genome-wide DNA methylation pattern in tomato indicated that, as in *A. thaliana*, there was a clear partition of the RdDM machinery between chromosome arms and pericentric heterochromatin. The *slnrpd1* and *slnrpe1* mutants had a dramatic loss in all mCHH subcontexts in the chromosome arms (where overall mCHH was down from 11% to 3%) but methylation remained at near wild type levels in the pericentric heterochromatin (Fig 5). These mutants also showed a mild decrease in CHG methylation in the arms (S13 Fig). The high level of residual CHH methylation in the pericentromere of these mutants indicates that tomato, like *A. thaliana*, has a CMT2-like pathway but that, rather than CAA/CTA, the preferred target sites are CAA and CAT. The other subcontext preferences for the putative CMT2-like methyltransferases in tomato are more continuous than those in *A. thaliana* and maize, with the presence of another C being disfavored.

### CMT2-like function of maize ZMET2 and ZMET5

**Fig 6:**
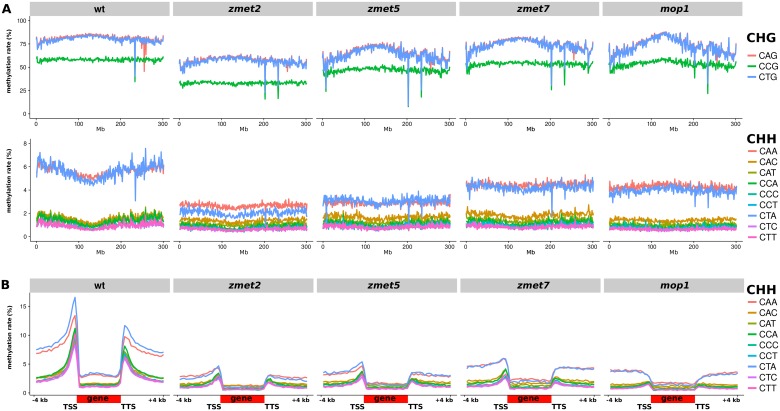
Maize DNA methylation in *CMT* and RdDM mutants. (A) CHG and CHH subcontext methylation along chromosome 1. (B) Average CHH subcontext methylation over genes. CHH islands [11] are clearly visible upstream of the transcriptional start site (TSS) and downstream of the transcription termination site (TTS), and depend both on RdDM and CMT methylation.

Maize does not have an AtCMT2 ortholog [5, 14, 22, 23] but there was preferential CHH methylation of CAA and CTA, as in *A. thaliana*. However the chromomethyltransferases encoded by *Zmet2* and *Zmet5* methylate cytosines in the CHH context as well as CHG [14], so we hypothesised that they mediate the differential subcontext methylation in these contexts. Consistent with this hypothesis both of the corresponding mutants had reduced mCHH along chromosome 1 that was most marked at CAA and CTA, in addition to lower CHG methylation than wild type (Fig 6A and S14 Fig). The *zmet2* mutation had a larger effect than *zmet5* at both CHG and CHH (Fig 6A and S14 Fig) and a particularly strong reduction at CCG and CTA. As in *A. thaliana* [13], there was some interdependence of CMT-dependent CHH methylation and RdDM: CAA and CTA methylation was reduced in similar ratios to other CHH contexts in the *zmet7* (homolog to *AtDRM2*) and *mop1* (ortholog to *AtRDR2*) RdDM mutants, and conversely the other CHH subcontexts (RdDM targets) had reduced methylation in *zmet2/5* mutants (Fig 6A and S14 Fig). This interdependence was similarly apparent at the gene-flanking CHH islands characteristic of maize [11], previously thought to be RdDM-dependent but also exhibiting a strong decrease in methylation in the *zmet2* and *zmet5* mutants (Fig 6B). The current maize transposon annotation is incomplete and does not allow a family-specific analysis of methylation patterns.

## Discussion

The conventional classification of DNA methylation in plant genomes in terms of CG, CHG and CHH sequence contexts reflects the action of various DNA methyltransferases associated with establishment and maintenance of epigenetic marks [4]. From this present analysis, however, we reveal that sequence subcontext in the DNA methylome is additionally informative about the partition of euchromatin and heterochromatin and the involvement of the DNA methyltransferases and H3K9 methyltransferases in these chromosomal domains. The partition of chromatin into pericentric (and possibly other types of) heterochromatin and euchromatic domains was most clearly evident in the dicot species *A. thaliana* and tomato (Fig 1). It is well known that the heterochromatin is the more methylated domain but we now show that at CHG motifs it has a lower ratio of mCCG/(mCAG or mCTG) than in the euchromatin (Figs 1 and 2) and that at CHH motifs there is a higher ratio of (mCAA-mCTA)/(other mCHH) (*A. thaliana)* or (mCAA-mCAT)/(other mCHH) (tomato) (Figs 1, 2 and 3).

Methylation at CCG in *A. thaliana* requires both MET1 and CMT3 (Fig 2) as in *Physcomitrella patens* [9], whereas other CHG contexts only require CMT3. In maize it is likely that ZMET2/5 and the MET1 orthologs are required for CCG methylation while CAG and CTG would require ZMET2/5 only. Yaari et al. proposed an explanatory model where CMT3 is unable to methylate the CGG motif (solely a substrate of MET1) on the strand opposite to CCG, and requires hemimethylation (mCGG) to methylate CCG [9]. However this hypothesis contradicts the molecular data on CMT3, which demonstrated that CMT3 efficiently methylates unmethylated substrates [24]. As an alternative we propose that MET1-mediated mCGG and CmCG recruits SUVH5/6, but not SUVH4, which would catalyse dimethylation of H3K9 and subsequent recruitment of CMT3 to methylate the first cytosine of CCG (S15 Fig). This model implies that the interaction between H3K9me2 and CHG methylation is very local, i.e. CHG methylation is controlled at the single-nucleosome level (or by the two adjacent nucleosomes only), which is consistent with the 167-bp periodicity of CHG methylation in the Arabidopsis genome [7].

A lower efficiency of the SUVH5/6-CMT3 feedback loop relative to the SUVH4-CMT3 loop would thus account for the lower methylation of CCG compared to CAG/CTG. Although trinucleotide contexts have not been taken into account, existing data on SUVH4/5/6 are consistent with our hypothesis: SUVH4 binds mCHG and mCHH much more strongly than mCG [25, 26], SUVH5 binds mCG/mCHG/mCHH with similar affinities [27], and SUVH6 prefers mCHG and mCHH to mCG but has not been tested on mCGG [25]. These differences in binding affinities may contribute to the locus-specificity that SUVH4, SUVH5 and SUVH6 exhibit [18]. Notably, the role of SUVH5/6 to the exclusion of SUVH4 in the regulation of *A. thaliana* rDNA loci [28] may be due to the high density of CCG/CGG duplexes in the 5’ external transcribed sequence (5’ ETS) of the 45S rDNA repeat.

The CHH methylation biases are influenced by various chromomethyltransferases. In *A. thaliana* (Fig 2) CMT2 preferentially methylates CAA and CTA. In tomato, there are three members of the CMT family [29, 30] but their activities have yet to be defined. Based on multiple independent CMT losses in eudicots and monocots, a recent analysis proposes that the different CMT clades (CMT*ϵ*, encompassing CMT1 and CMT3, and CMT2) may have overlapping functions [31]. In agreement with this, in maize with no CMT2 ortholog, it is likely that ZMET2 and ZMET5 from the CMT*ϵ* clade share roles that are separated in the *A. thaliana* CMT2 and CMT3. A similar situation may apply in rice in which there are three as yet uncharacterised *CMT* genes [32].

The subcontext differences in CHH methylation may be due to intrinsic affinities of the CMT proteins, to the affinities of factors that mediate CMT recruitment as was the case for CMT3 and SUVH4/5/6, or to a combination of both mechanisms. It is likely that at least certain members of the SUVH family of H3K9 methyltransferases have an affinity for methylated CTA/CAA motifs in *A. thaliana*, which would establish a positive feedback loop with CMT2-dependent DNA methylation similar to the well established feedback loop between H3K9 methylation by SUVH4/KYP and DNA methylation in CHG contexts by CMT3 [24]. Furthermore, differential subcontext methylation may be informative to methylation readers: recognition of heterochromatic, CMT2-controlled mCAA/mCTA is likely to trigger different responses than binding to RdDM-controlled mCTT sites in a more open chromatin environment. It is possible that the CMTs evolved these affinities in part to control CG sites that would mutate via deamination of methylcytosine: mCG deamination would create a CAN site on the opposite strand, while deamination of mCAG or mCTG would give rise to CTA and CAA sites. This might be a way of maintaining methylation-dependent silencing of loci despite their tendency to lose cytosines.

Contrasting with the motif biases and heterochromatic substrates of chromomethyltransferases, RdDM is mostly active in chromosome arms and does not have obvious sequence context bias. This sequence-independence likely reflects the fact that the DRM2 DNA methyltransferase is guided by RNA as opposed to the protein-DNA interactions of the maintenance DNA methyltransferases.

It is striking that growth and development of tomato is greatly affected by perturbations of DNA methylation, in this case the RdDM pathway, as in rice and maize [14, 33]. By contrast, in *A. thaliana*, various methylation mutants including *nrpd1* and *nrpe1* are fully viable and exhibit near normal development [34, 35]. It is likely that differential effects of RdDM mutations between species are connected to transposons and their epigenetic influence on the expression of adjacent genes. RdDM would have a relatively small effect on genes adjacent to elements like LINE/L1 (Fig 2E) at which methylation persists in a CMT2-dependent manner whereas, at elements like RathE1 that are subject to RdDM, the effect would be much greater. Until now it was necessary to use mutants to identify genes that are likely to be affected by RdDM but now, in the light of our analysis, it will be possible to screen methylomes for genes likely to be affected by RdDM, where CHH methylation is independent of sequence context.

Our comprehensive analysis of methylation in trinucleotide contexts in *A. thaliana*, tomato, maize and rice has revealed additional complexity in the plant methylomes but it could just be a first step. Although analysis of trinucleotides does capture the largest differences while keeping the number of combinations manageable, extending to surrounding nucleotides may refine our understanding of methyltransferase and methyl-binding proteins affinities. In principle, there could be GC maintenance methylases in addition to the well-characterised enzymes with CG substrates. There could also be methyltransferases acting at any symmetric C(H)_*n*_G or G(H)_*n*_C patterns (in which *n* ≥ 1) provided that the enzyme, either as a monomer or multimer, could span the cytosines on the two DNA strands of these motifs. Extended analyses of existing and future methylome datasets will be informative about these possibilities.

## Materials and Methods

### CRISPR-Cas gene editing in tomato

Mutants were obtained by stably transforming tomato plants expressing Cas9 and pairs of sgRNAs. Pairs of sgRNAs were designed to be unique to the gene of interest, upstream of a NGG Protospacer Adjacent Motif (PAM), in an exon towards the 5’ region of the predicted transcript and separated by 200-300 nt. We used NCBI GNOMON33088049 as *SlNRPD1* gene model, and ITAG Solyc01g096390.2.1 as *SlNRPE1* gene model. The sgRNAs were amplified from plasmid pICH86966::AtU6p::sgRNA-PDS (Addgene plasmid 46966) with the custom forward primers “sg fw” and the common reverse primer “sg rv” (sequences in S2 Table), and placed under the AtU6p promoter by cut-ligation with the level 0 construct pICSL01009::AtU6p and a level 1 destination vector pICH47751 (for the first sgRNA of the pair) or pICH47761 (for the second) [36]. A second cut ligation of the obtained plasmid with pICH47732::NOSp::NPTII-OCST, pICH47742::35S::Cas9-NOST, the pICH41780 linker and the pAGM4723 level2 destination vector. The final plasmid was transformed into *Solanum lycopersicum* cv. M82, and a similar plasmid without sgRNAs was transformed as control.

Sterile seeds were germinated on 1/2 strength Murashige-Skoog medium, 1X Nitsch & Nitsch vitamins, 0.8% agar, 1.5% sucrose, pH 6. Cotyledons from 8-day-old plants were cut in two and submerged in a solution of Agrobacterium in MS, 3% sucrose at OD_600_ = 1.5. The explants were then quickly dried on Whatman paper and placed on a plate without selection under low light (1X MS, 1X Nitsch & Nitsch vitamins, 0.6% agarose, 3% sucrose, 100 mg.l^−1^ myo-inositol, 0.5 mg.l^−1^ 2,4-D, 0.1 mg.l^−1^ kinetin, pH 5.7). After 48 h the explants were transferred to a selection plate (1X MS, 1X Nitsch & Nitsch vitamins, 0.4% agargel, 2% sucrose, 100 mg.l^−1^ myo-inositol, 2 mg.l^−1^ zeatin, 100 mg.l^−1^ kanamycin, 320 mg.l^−1^ timentin, pH 6), and this was repeated every two weeks until regenerating shoots started to push the lid. The shoots were then transferred to jars with selection media supplemented with 250 mg.l^−1^ cefotaxime. After five weeks the shoots were transferred to rooting media (1/2 strength MS medium, 1X Nitsch & Nitsch vitamins, 0.225% gelrite, 0.5% sucrose, 50 mg.l^−1^ kanamycin, 320 mg.l^−1^ timentin, pH 6). Regenerants with well-developed roots were then transferred to peat bags and grown under high humidity until they could be transferred to M3 compost and grown under normal conditions. Regions targeted by sgRNAs were then amplified from genomic DNA, cloned and Sanger sequenced.

### MethylC-Seq

DNA was extracted from 100 mg of leaf tissue using the Puregene kit (QIAGEN). Bisulfite library preparation was performed with a custom protocol similar to [37]. 1.2 *μ*g DNA was sonicated on a Covaris E220 to a target size of 400 bp and purified on XP beads (Ampure, ratio 1.8X). DNA was end-repaired and A-tailed using T4 DNA polymerase and Klenow Fragment (NEB) and purified again using XP beads (ratio 1.8X). Methylated Illumina Y-shaped adapters for paired-end sequencing were ligated using Quick-Stick Ligase (Bioline). 450 ng of purified (ratio 1.8X), adapter-ligated DNA was bisulfite-converted using the EZ DNA Methylation-Gold Kit (Zymo Research) according to the manufacturer’s instructions. DNA was barcoded using 12 cycles of PCR amplification with KAPA HiFi HotStart Uracil+Ready Mix (Kapabiosystems) with PE1.0 and custom index primers (courtesy of the Sanger Institute). Pooled libraries were sequenced to a depth of about 5X on a HiSeq 2500 125PE.

Sequences were trimmed and filtered with Trim Galore! (default parameters), then mapped onto the respective genomes (TAIR10 for *A. thaliana*, Heinz SL2.50 for tomato, RefGen B73 v3 for maize, Oryza indica ASM465 v1.28 for rice) using Bismark v0.14.5 [38] with option -N 1 (and -X 1500 for paired-end data). Reads were deduplicated with bismark-deduplicate and methylation calls were extracted using Bismark methylation_extractor (with option −*r*2 2 for paired-end reads).

Genome-wide cytosine reports were generated with Bismark coverage2cytosine [38] and average methylation in trinucleotide context calculated in 500 kb (for *A. thaliana*) or 1 Mb bins (non-weighted mC/(mC+C)). Average profiles over genes and transposons were calculated from the cytosine reports with segmentSeq v2.4.0 [39], using the TAIR10, ITAG2.4, AGP v3.31, 9311-glean-gene gene annotations, and TAIR10, tomato LTR transposons [40], AGP v3.31 (repeat regions larger than 1 kb), 9311-repeat-Repbase transposon annotations. Average plots for the *A. thaliana* transposon families are based on the annotation by Buisine et al. [19].

### sRNA-Seq

sRNAs were cloned from 10 *μ*g total RNA using the Illumina TruSeq Small RNA cloning kit and libraries were indexed during the PCR step (12 cycles) according to the manufacturer’s protocol. Gel size-selected, pooled libraries were sequenced on a HiSeq 2000 50SE. Sequences were trimmed and filtered with Trim Galore! (with the adapter parameter -a TGGAATTCTCGGGTGCCAAGG) and reads were mapped without mismatches and clustered on Heinz genome SL2.50 using the ShortStack software v2.1.0 [41]. sRNA counts on the defined loci were analyzed with DESeq2 v1.8.1 [42]. Normalisation factors from the 20–22-nt sRNAloci were used to normalise counts on 24-nt loci.

### Accession numbers

We used *A. thaliana* bisulfite data (GSE39901) generated by Stroud et al. [13]; maize bisulfite data (GSE39232) by Li et al. [14]; rice bisulfite data (GSE38480) by Chodavarapu et al. [15]; human bisulfite data (SRR901864 and SRR921754) from Lister et al. [43]. Bisulfite and small RNA sequencing data for tomato are available under study accession SRP081115.

## Supporting Information

S1 Fig DNA methylation in trinucleotide contexts for all *A. thaliana* (Col-0) chromosomes. (TIF)

S2 Fig DNA methylation in trinucleotide contexts for all tomato (M82) chromosomes. (TIF)

S3 Fig DNA methylation in trinucleotide contexts for all maize (B73) chromosomes. (TIF)

S4 Fig DNA methylation in trinucleotide contexts for all rice (indica) chromosomes. (TIF)

S5 Fig Average CG DNA methylation over genes (A) and transposons (B) in trinucleotide contexts for the four species under study. (TIF)

S6 Fig DNA methylation in trinucleotide contexts in human (*Homo sapiens*) brain (middle frontal gyrus) and ES cells. Chromosome 1 in 1 Mbp bins (libraries from [43]).
(TIF)

S7 Fig DNA methylation in trinucleotide contexts for *A. thaliana* Col-0 after perfect alignment of reads (with option --score_min L, 0, 0, no mismatch allowed). (TIF)

S8 Fig Trinucleotide motif distribution in *A. thaliana*. (A) Trinucleotide density along chromosome 1. (B) Motif densities on chromosomes, genes and transposable elements.
(TIF)

S9 Fig DNA methylation in trinucleotide contexts along chromosome 1 for *A. thaliana* Col-0 (wt) and the triple demethylase mutant *ros1/dml2/dml3* (*rdd*). (TIF)

S10 Fig CHG and CHH methylation in *A. thaliana* mutants relative to wt. Ratio of mutant over wt methylation rate along chromosome 1.
(TIF)

S11 Fig Density of methylation ratio at individual CHH sites. Sites of chromosome 1 in *A. thaliana* with sequencing depth of at least 8, in pericentric heterochromatin (13–16 Mb) and chromosome arms (0–10 Mb and 20–30Mb).
(TIF)

S12 Fig CHH subcontext methylation average over *A. thaliana* transposons superfamilies. Annotation from [19].
(TIF)

S13 Fig Decreased CHG methylation in the chromosome arms of tomato RdDM mutants. Chromosome 1 is shown.
(TIF)

S14 Fig Maize DNA methylation in *CMT* and RdDM mutants. CHG and CHH subcontext methylation along chromosome 1, relative to wt methylation (B73).
(TIF)

S15 Fig Model of methylation at CAG/CTG and CCG/CGG sites. (A) Current model of CAG/CTG methylation. SUVH4/KYP is the main H3K9 histone methyltransferase, and mCAG/mCTG is efficiently maintained after replication. (B) Proposed model of CCG/CGG methylation, depending on MET1 and SUVH5/6. The lower efficiency of SUVH5/6 compared to SUVH4 would account for the lower CCG methylation level observed in heterochromatin, compared to CAG/CTG methylation. Because CG methylation is efficiently maintained by MET1 independently of H3K9me2, loss of mCCG after one replication may be rescued at a later replication. Additionally, CCG sites in close proximity to SUVH4-bound mCAG/mCTG may experience better-maintained methylation than isolated CCG sites thanks to increased CMT3 recruitment by SUVH4-mediated H3K9me2.
(TIF)

S1 Table Bisulfite conversion rates as determined from the *A. thaliana* chloroplast. (PDF)

S2 Table Oligonucleotides used in this study. (PDF)

S1 Dataset Table of tomato sRNA loci and counts in wild-type and RdDM mutants. (TXT)
