# Supplementary figures and images for: DNA Methylation Signatures of the Plant Chromomethyltransferases
Source: PLoS Genet. 2016 Dec 20;12(12):e1006526. doi: 10.1371/journal.pgen.1006526 (PMC5221884; doi:10.1371/journal.pgen.1006526)

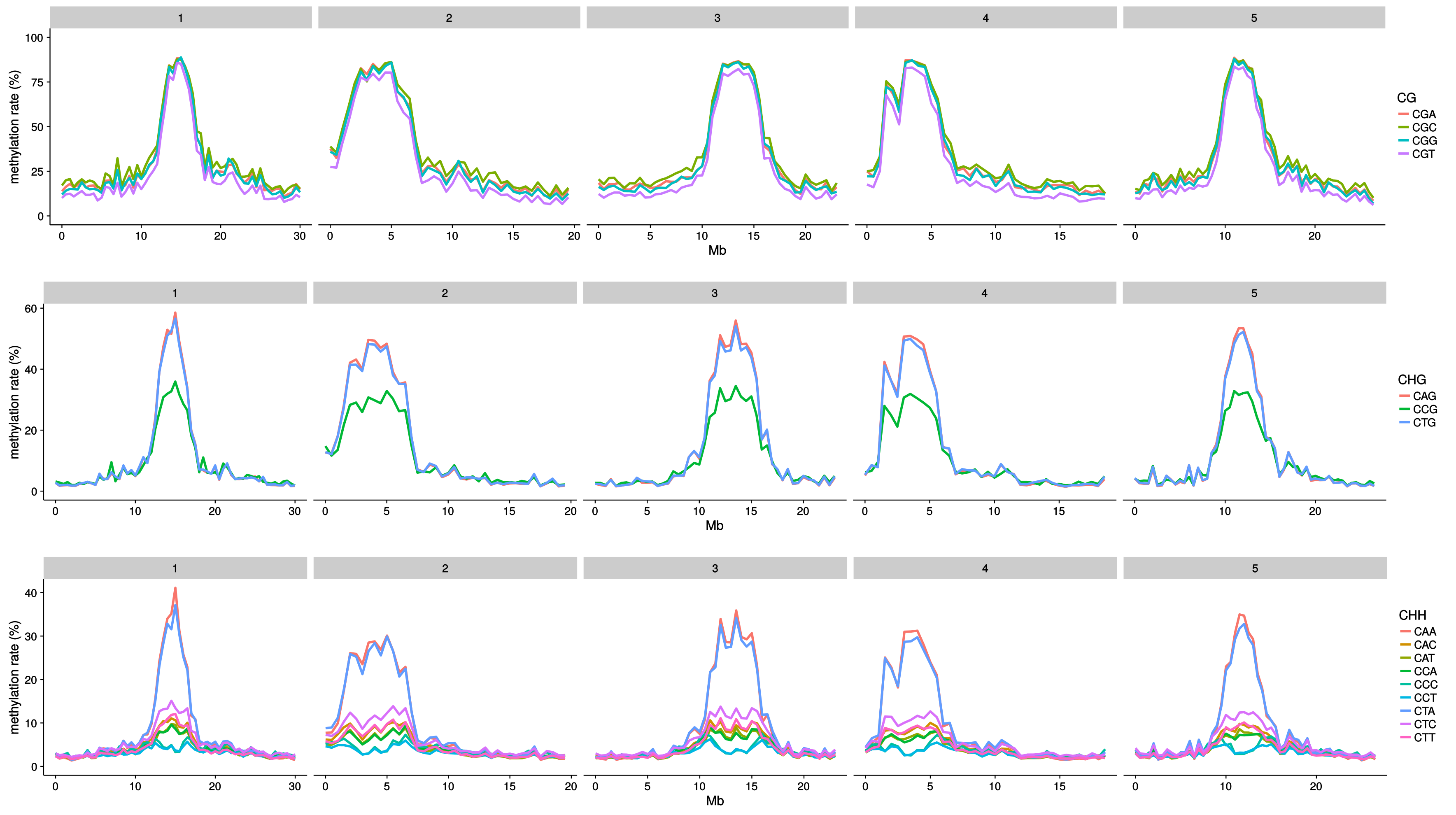

Supplement: S1 Fig — (TIF) [file pgen.1006526.s001.tif]

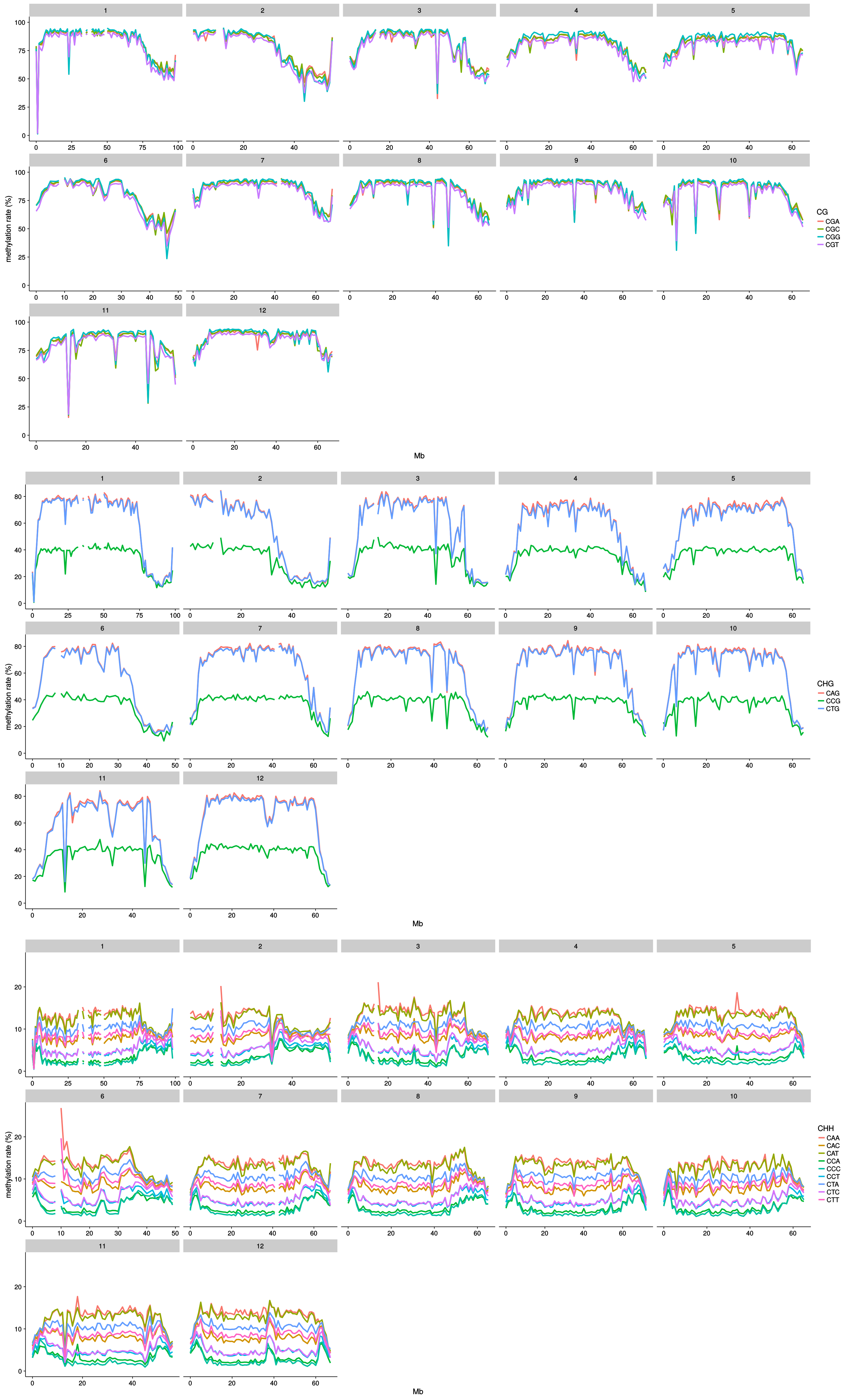

Supplement: S2 Fig — (TIF) [file pgen.1006526.s002.tif]

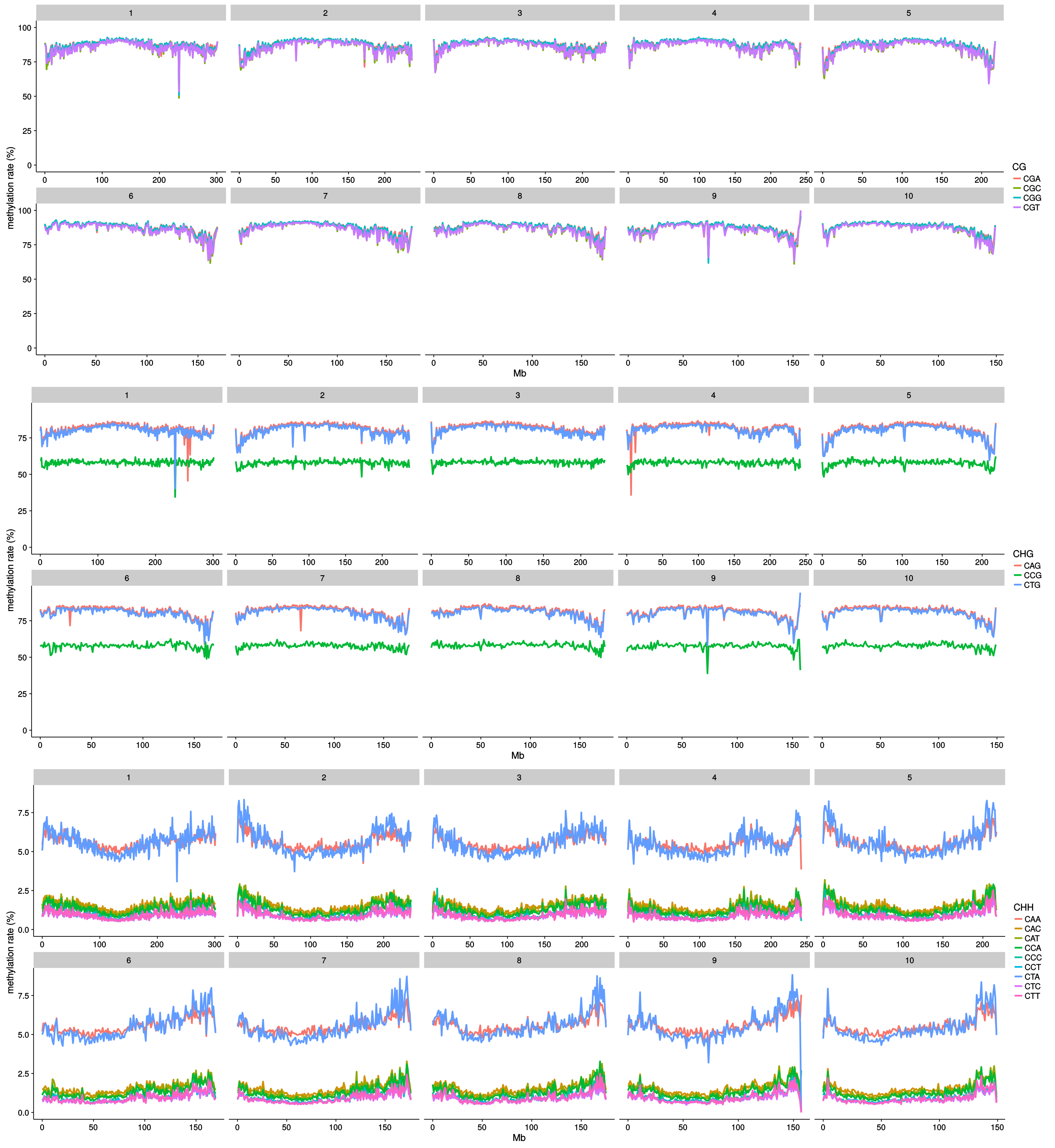

Supplement: S3 Fig — (TIF) [file pgen.1006526.s003.tif]

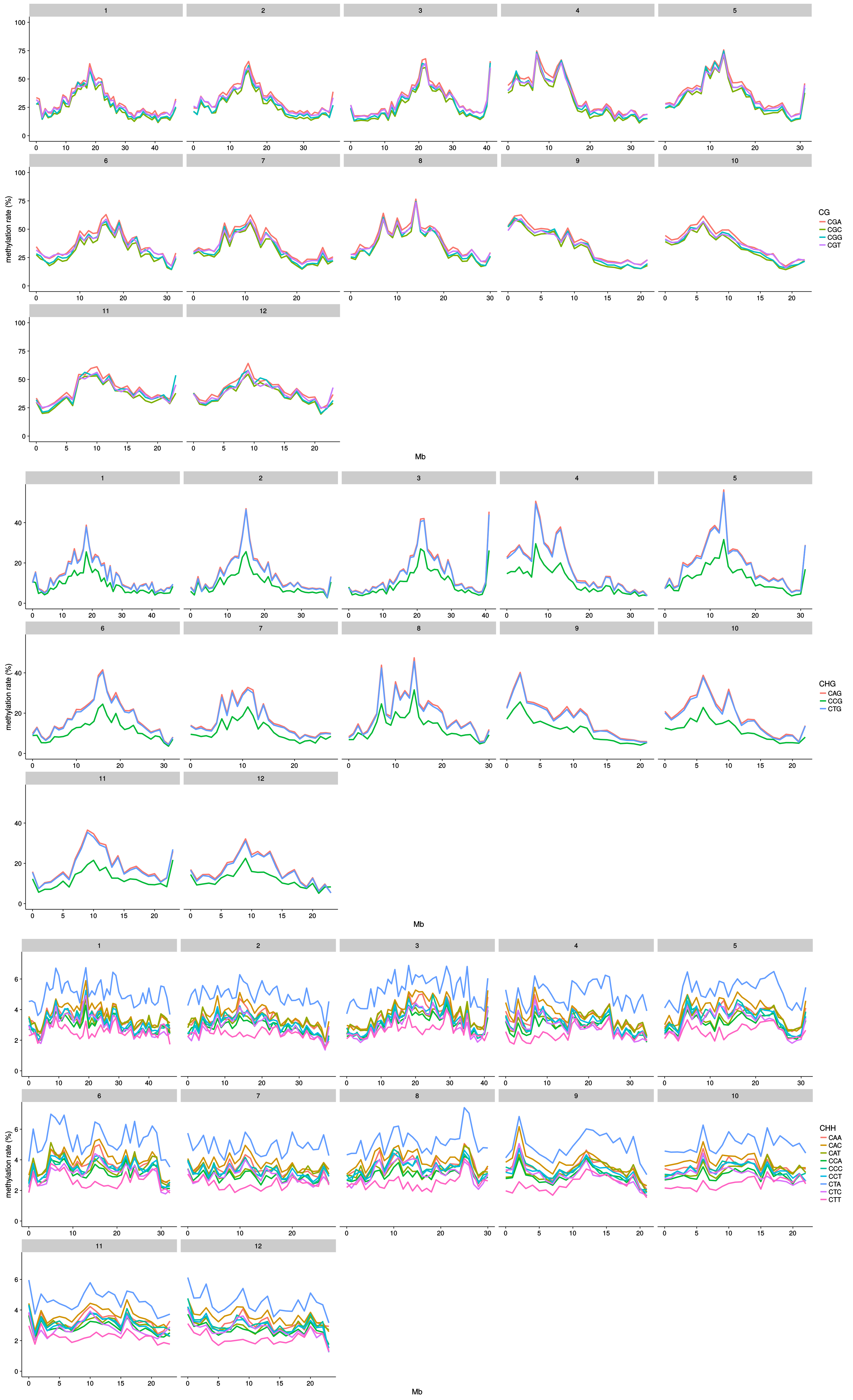

Supplement: S4 Fig — (TIF) [file pgen.1006526.s004.tif]

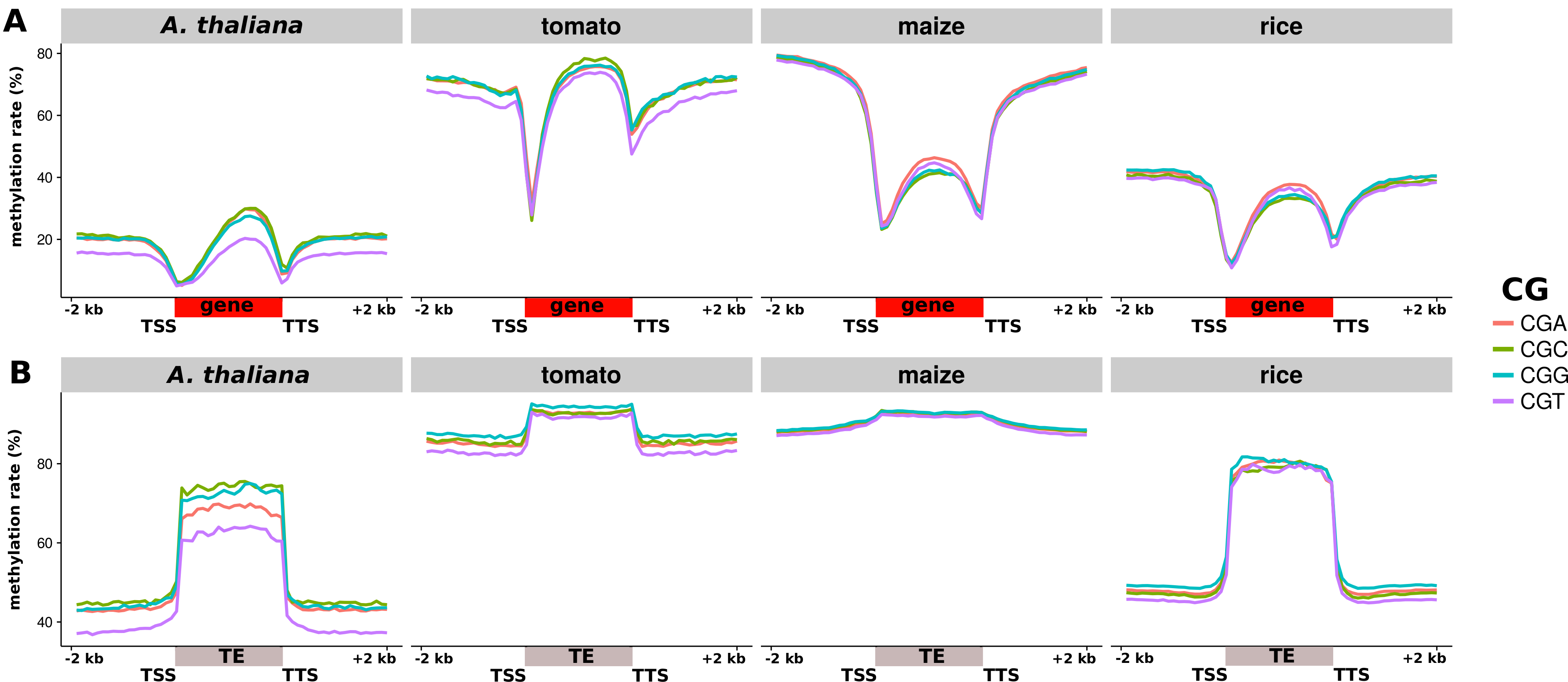

Supplement: S5 Fig — (TIF) [file pgen.1006526.s005.tif]

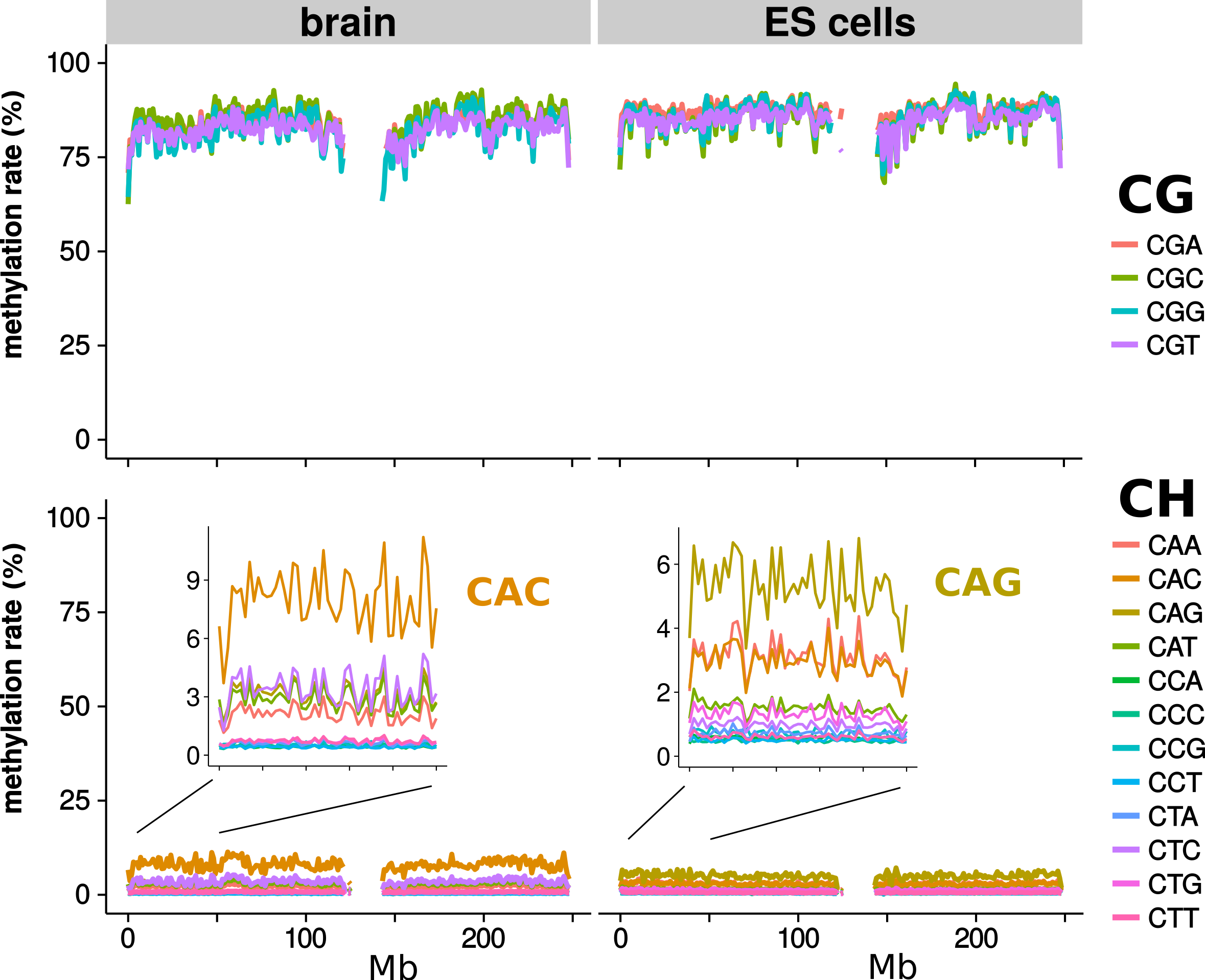

Supplement: S6 Fig — Chromosome 1 in 1 Mbp bins (libraries from [43]). (TIF) [file pgen.1006526.s006.tif]

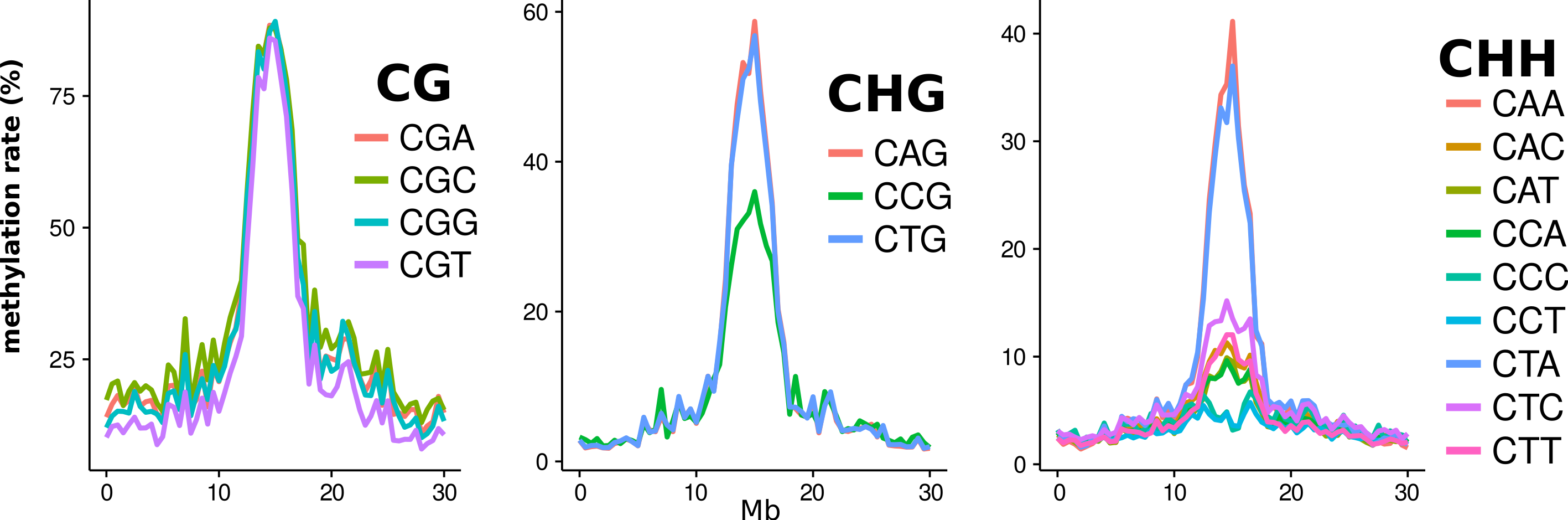

Supplement: S7 Fig — (TIF) [file pgen.1006526.s007.tif]

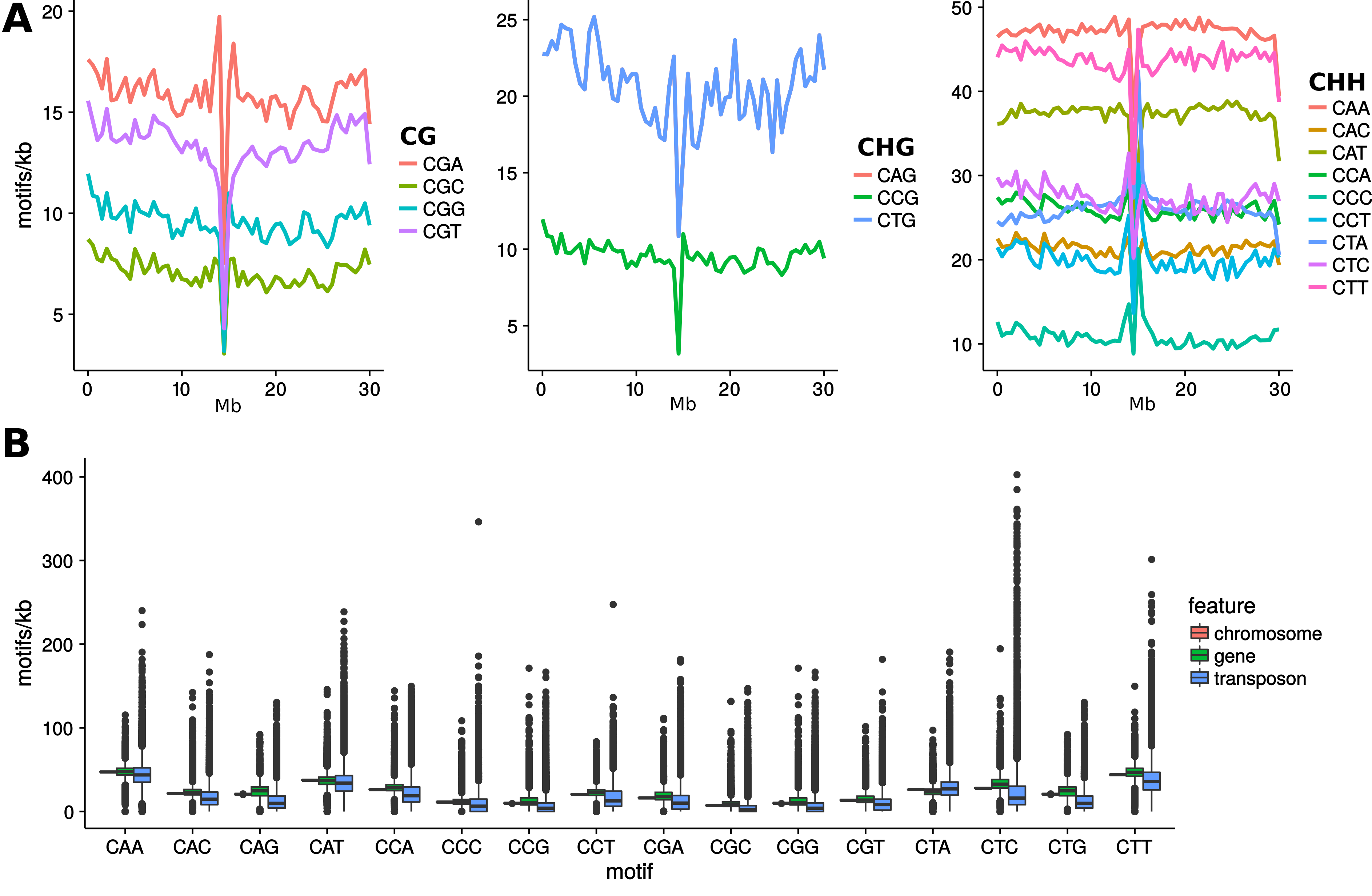

Supplement: S8 Fig — (A) Trinucleotide density along chromosome 1. (B) Motif densities on chromosomes, genes and transposable elements. (TIF) [file pgen.1006526.s008.tif]

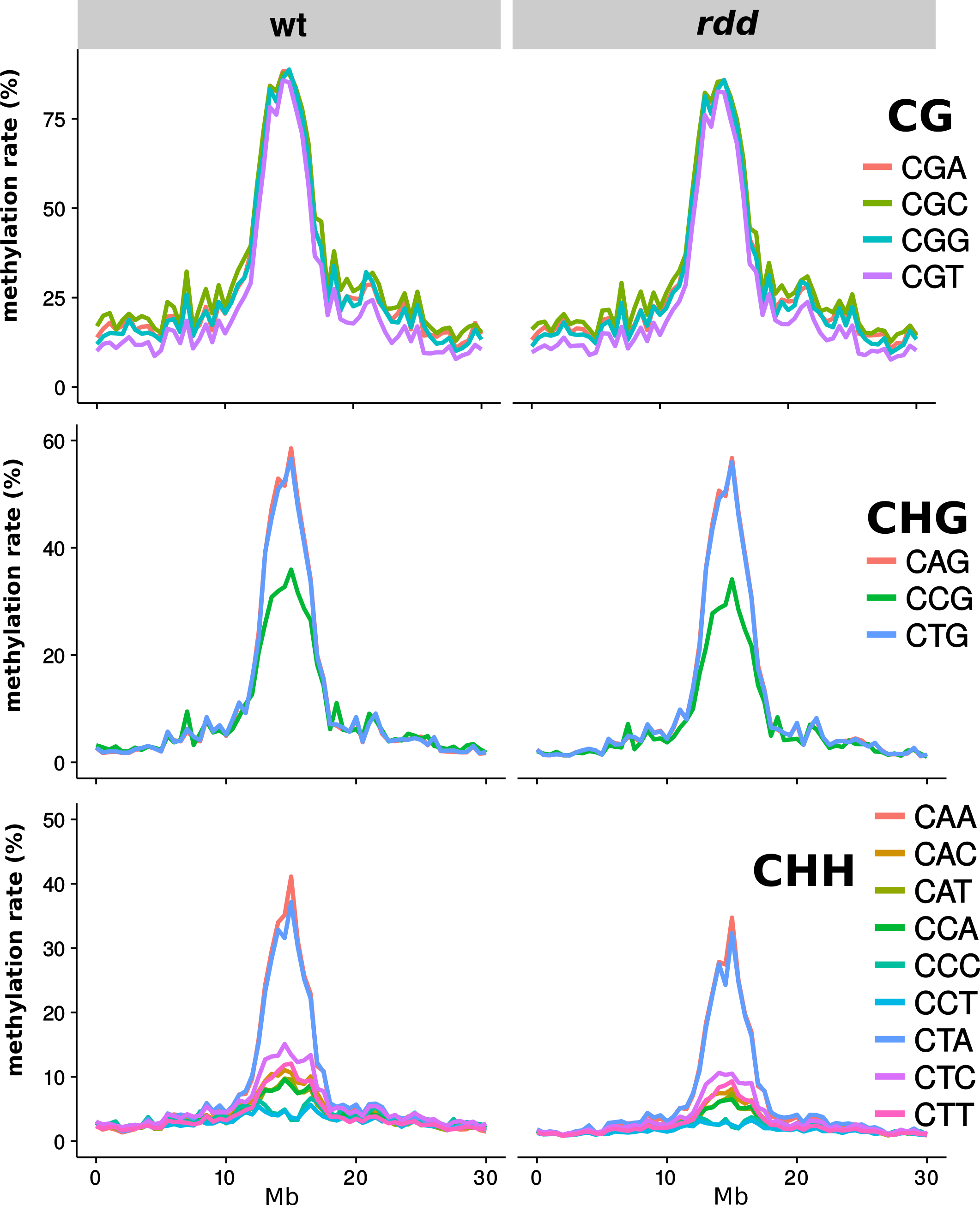

Supplement: S9 Fig — (TIF) [file pgen.1006526.s009.tif]

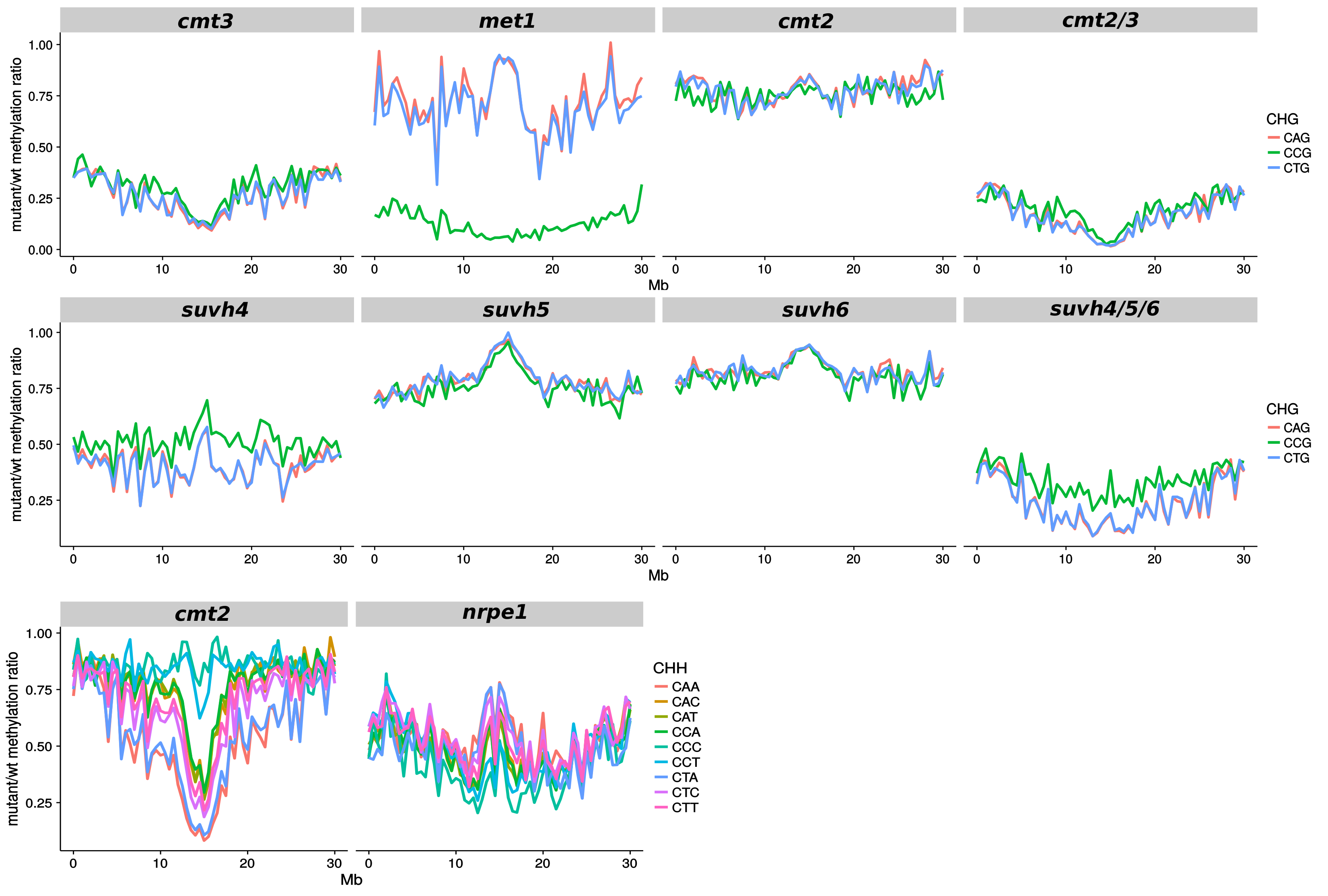

Supplement: S10 Fig — Ratio of mutant over wt methylation rate along chromosome 1. (TIF) [file pgen.1006526.s010.tif]

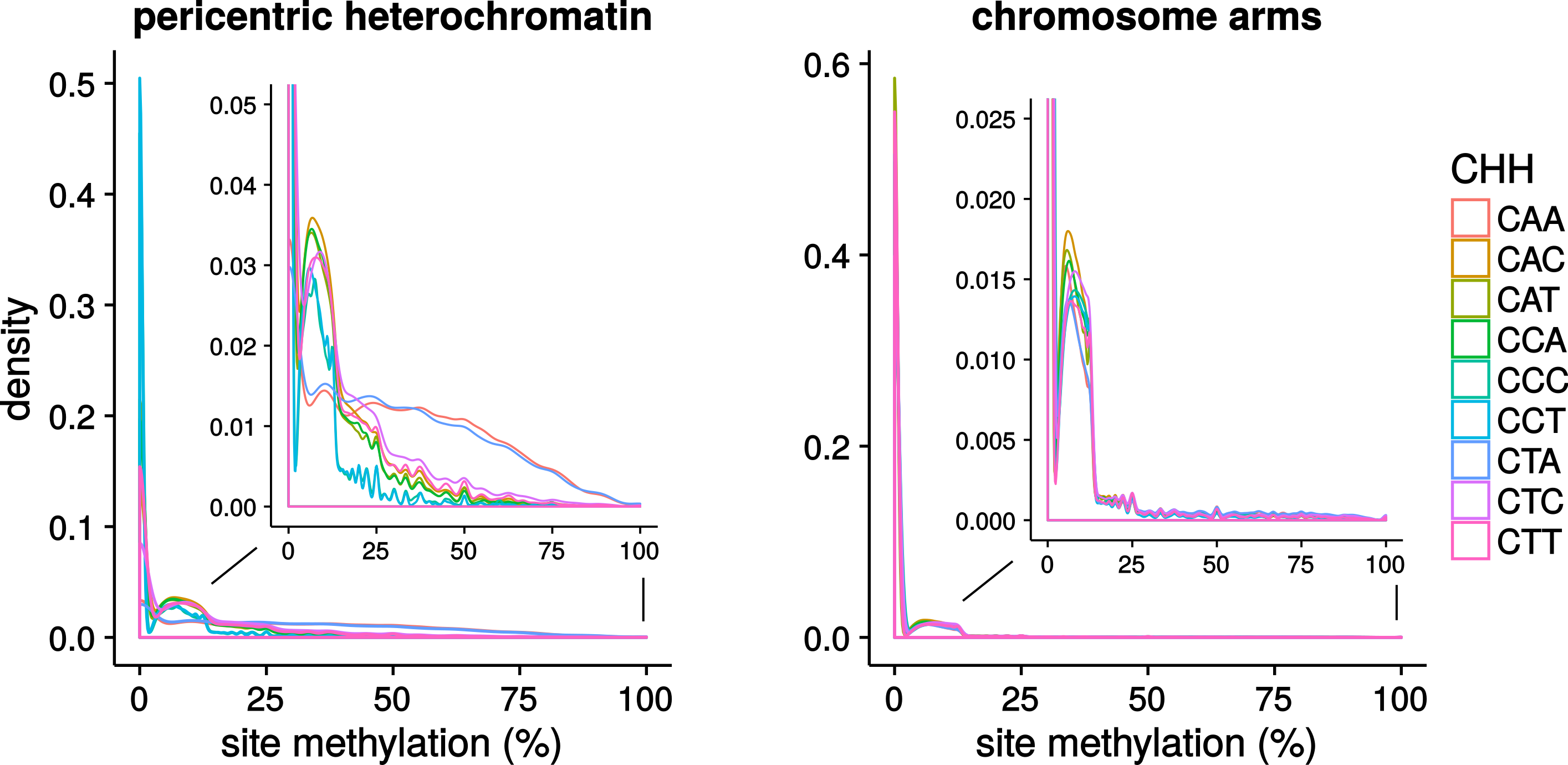

Supplement: S11 Fig — Sites of chromosome 1 in A. thaliana with sequencing depth of at least 8, in pericentric heterochromatin (13–16 Mb) and chromosome arms (0–10 Mb and 20–30Mb). (TIF) [file pgen.1006526.s011.tif]

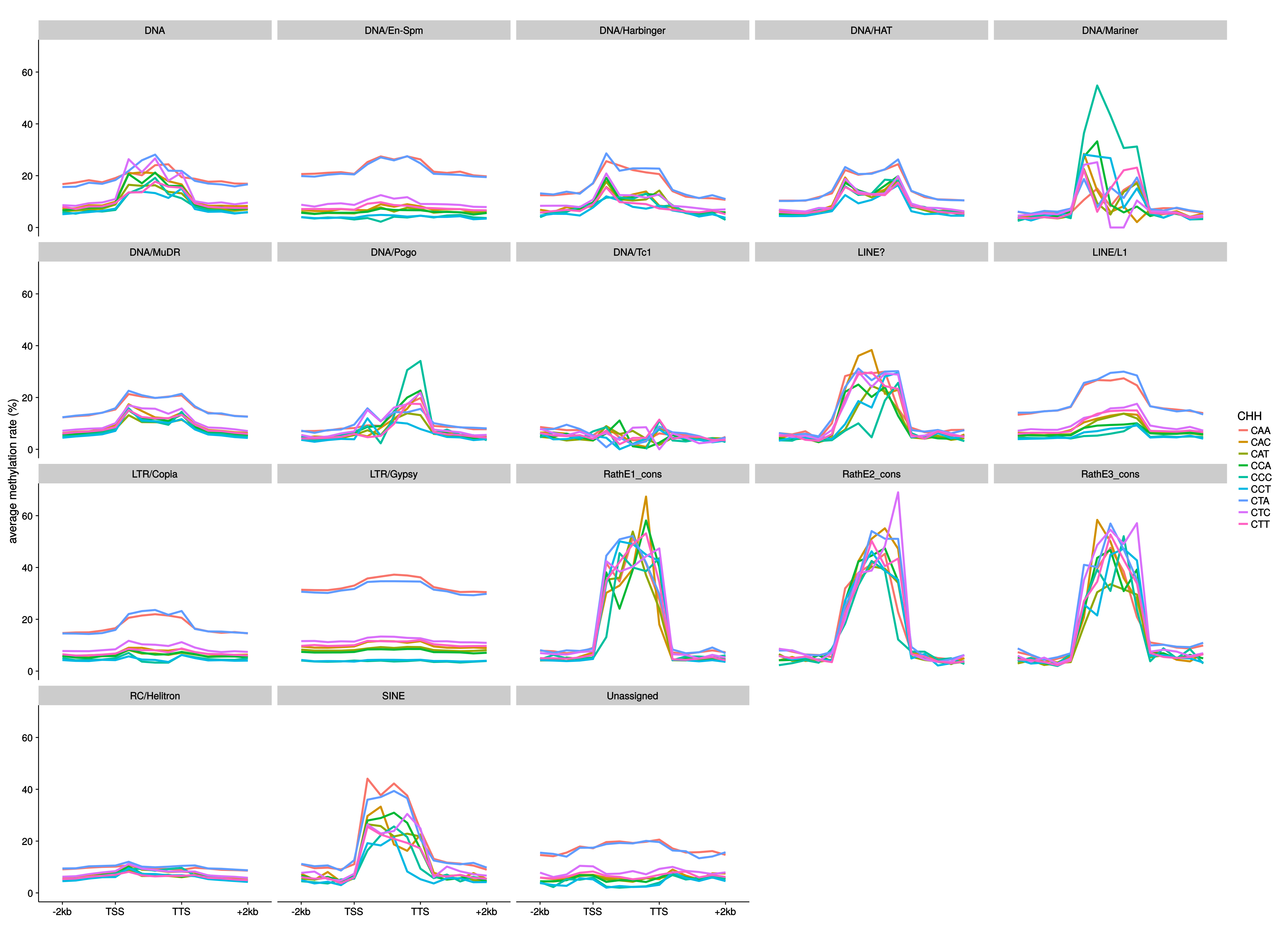

Supplement: S12 Fig — Annotation from [19]. (TIF) [file pgen.1006526.s012.tif]

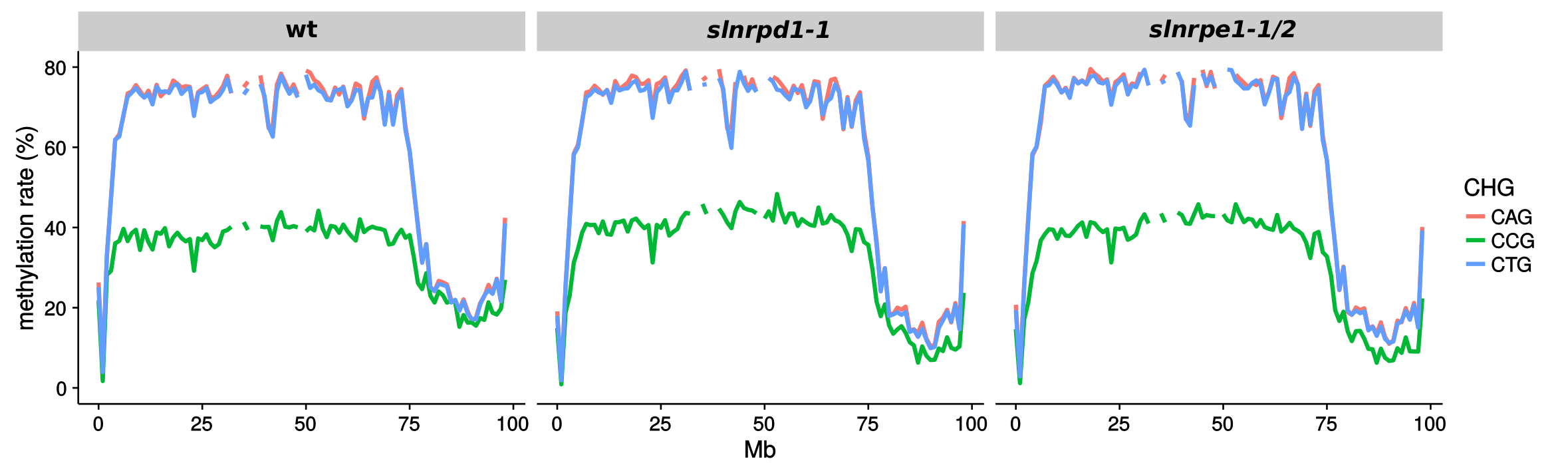

Supplement: S13 Fig — Chromosome 1 is shown. (TIF) [file pgen.1006526.s013.tif]

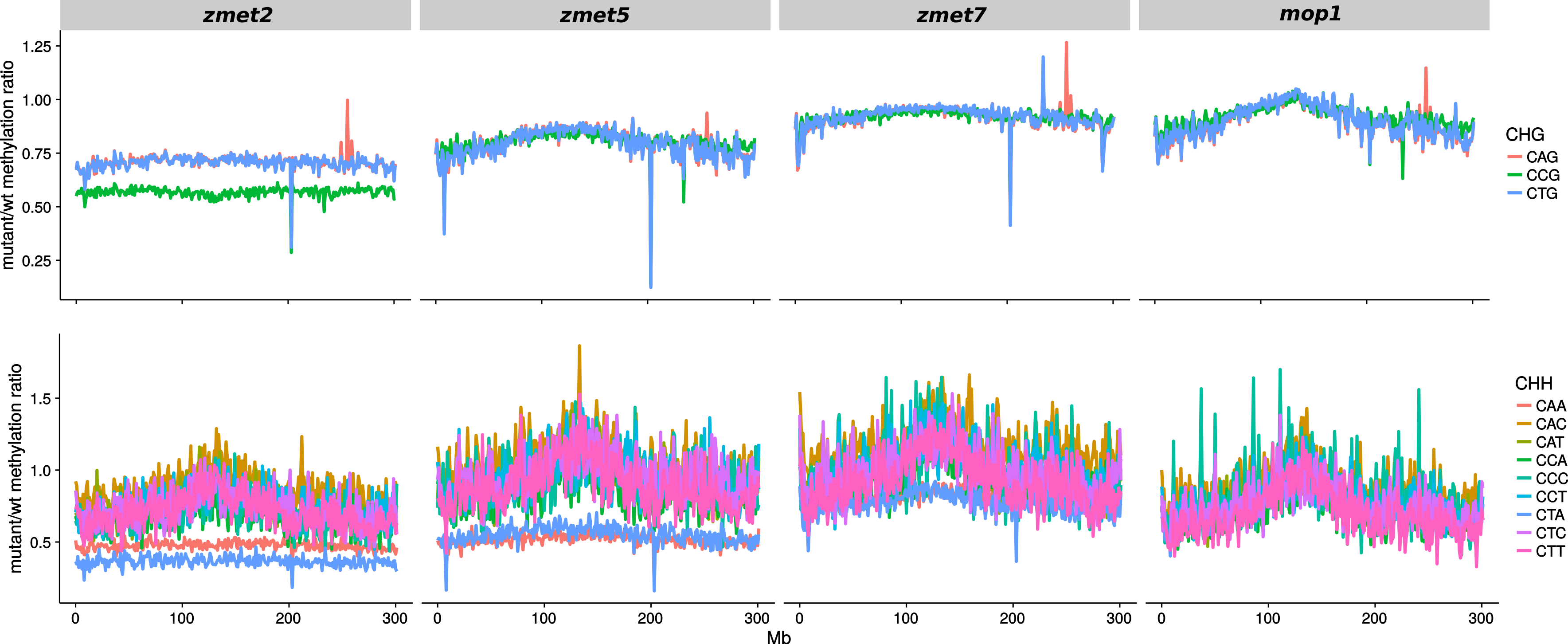

Supplement: S14 Fig — CHG and CHH subcontext methylation along chromosome 1, relative to wt methylation (B73). (TIF) [file pgen.1006526.s014.tif]

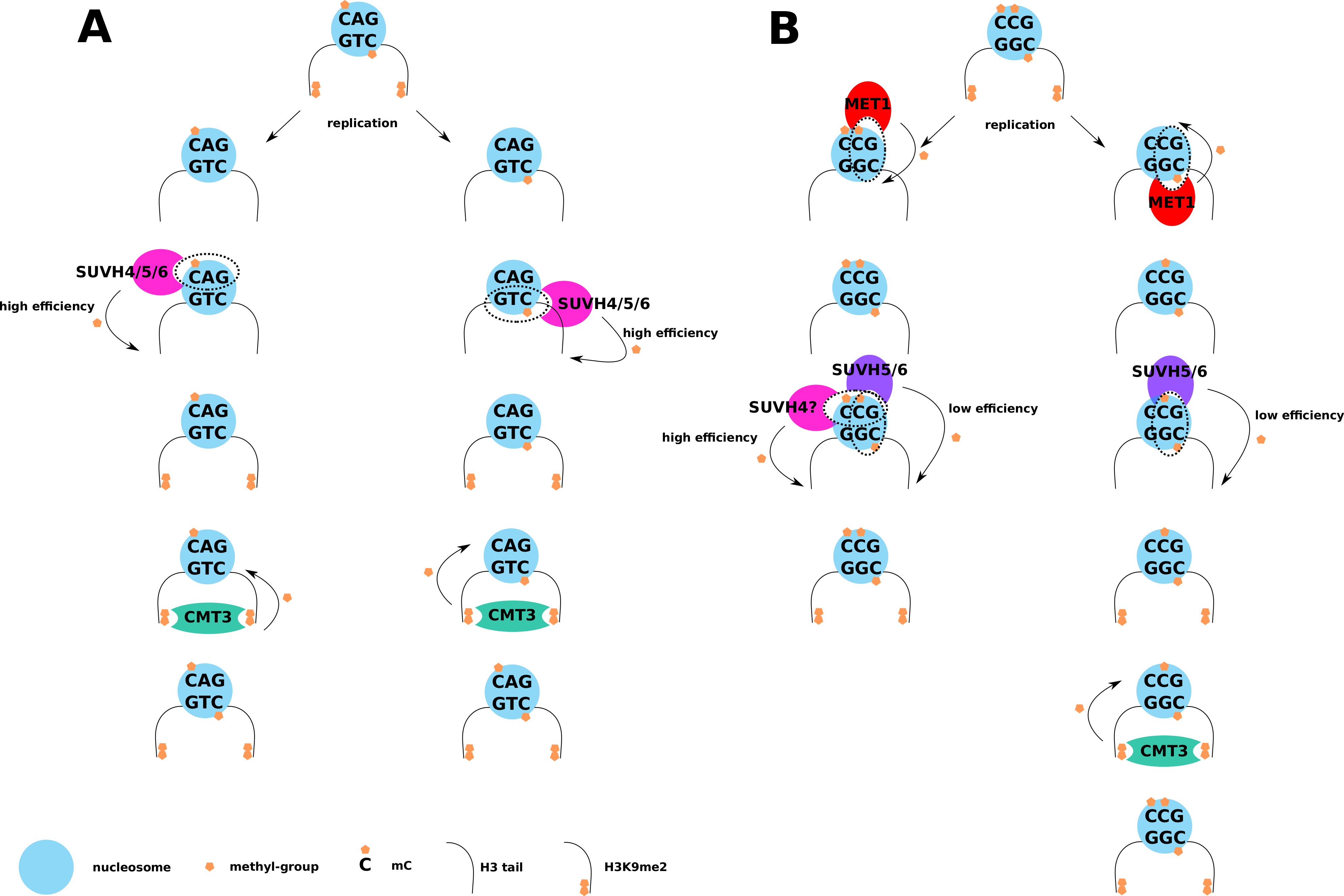

Supplement: S15 Fig — (A) Current model of CAG/CTG methylation. SUVH4/KYP is the main H3K9 histone methyltransferase, and mCAG/mCTG is efficiently maintained after replication. (B) Proposed model of CCG/CGG methylation, depending on MET1 and SUVH5/6. The lower efficiency of SUVH5/6 compared to SUVH4 would account for the lower CCG methylation level observed in heterochromatin, compared to CAG/CTG methylation. Because CG methylation is efficiently maintained by MET1 independently of H3K9me2, loss of mCCG after one replication may be rescued at a later replication. Additionally, CCG sites in close proximity to SUVH4-bound mCAG/mCTG may experience better-maintained methylation than isolated CCG sites thanks to increased CMT3 recruitment by SUVH4-mediated H3K9me2. (TIF) [file pgen.1006526.s015.tif]
