# Supplementary material for: DNA Methylation Signatures of the Plant Chromomethyltransferases
Source: PLoS Genet. 2016 Dec 20;12(12):e1006526. doi: 10.1371/journal.pgen.1006526 (PMC5221884; doi:10.1371/journal.pgen.1006526)
Supplement: S1 Table — (PDF) [file pgen.1006526.s016.pdf]

S1 Table: Bisulfite conversion rates (%) as determined from the *A. thaliana* chloroplast

**subcontext**	**Col-0_rep1**	**Col-0_rep2**
CAA	98.5	99.2
CAC	97.5	98.9
CAG	98.4	99.2
CAT	98	99.1
CCA	97.8	99.1
CCC	97.7	99.1
CCG	98.1	99.2
CCT	98	99.1
CGA	98.4	99.3
CGC	97.8	99.1
CGG	98.5	99.2
CGT	98.3	99.2
CTA	98.5	99.3
CTC	97.7	99
CTG	98.2	99.2
CTT	98.2	99.1
