# Supplementary material for: DNA Methylation Signatures of the Plant Chromomethyltransferases
Source: PLoS Genet. 2016 Dec 20;12(12):e1006526. doi: 10.1371/journal.pgen.1006526 (PMC5221884; doi:10.1371/journal.pgen.1006526)
Supplement: S2 Table — (PDF) [file pgen.1006526.s017.pdf]

S2 Table: Oligonucleotides used in this study

name	sequence
Slnrpd1 sg fw1	tgtggtctcaATTGATCCTAATGCCCCTGCTACgttttagagctagaaatagcaag
Slnrpd1 sg fw2	tgtggtctcaATTGGTGATCCTAACATAGAGCTgttttagagctagaaatagcaag
Slnrpe1 sg fw1	tgtggtctcaATTGGCATACATTTCTACGACCTgttttagagctagaaatagcaag
Slnrpe1 sg fw2	tgtggtctcaATTGTTTTGGAGCTTTTTGCTGTgttttagagctagaaatagcaag
sg rv	tgtggtctcaAGCGTAATGCCAACTTTGTAC
